# Participants’ understanding of a randomized controlled trial (RCT) through informed consent procedures in the RCT for breast cancer screening, J-START

**DOI:** 10.1186/1745-6215-15-375

**Published:** 2014-09-25

**Authors:** Yoko Narikawa Shiono, Ying-Fang Zheng, Masahiro Kikuya, Masaaki Kawai, Takanori Ishida, Shinichi Kuriyama, Noriaki Ohuchi

**Affiliations:** Division of Surgical Oncology J-START research unit, Tohoku University Graduate School of Medicine, 2-1, Seiryo-machi, Aoba-ku, Sendai Japan; Division of Molecular Epidemiology, Tohoku University Graduate School of Medicine, 2-1, Seiryo-machi, Aoba-ku, Sendai Japan; Department of Advanced Breast Cancer Imaging, Tohoku University Graduate School of Medicine, 2-1, Seiryo-machi, Aoba-ku, Sendai Japan; Department of Preventive Medicine and Epidemiology, Tohoku Medical Megabank Organization, Tohoku University, 2-1, Seiryo-machi, Aoba-ku, Sendai Japan; Department of Breast Oncology, Miyagi Cancer Centre Hospital, 47-1 Nodayama, Medeshima-Shiode, Natori, Miyagi Japan; Division of Molecular Epidemiology, Environment and Genome Research Centre, Tohoku University Graduate School of Medicine, 2-1, Seiryo-machi, Aoba-ku, Sendai Japan; Division of Disaster Public Health, International Research Institute for Disaster Science, Tohoku University Graduate School of Medicine, 2-1, Seiryo-machi, Aoba-ku, Sendai Japan

**Keywords:** Breast cancer screening, participant understanding, RCT, informed consent

## Abstract

**Background:**

It is often difficult to enrol healthy volunteers into a randomized controlled trial (RCT) as there are barriers to participants’ proper understanding of a trial. This study aimed to evaluate degrees of understanding of the informed consent (IC) process among healthy volunteers who participated in an RCT. Additionally, factors associated with degree of understanding were investigated.

**Methods:**

The J-START (the Japan STrategic Anti-cancer Randomized controlled Trial) is an RCT investigating the effectiveness of ultrasonography screening for breast cancer in women aged 40 to 49 years. To evaluate participants’ understanding of the J-START, we administered questionnaires to 376 Japanese women on the day of enrolment at five study sites across Japan. The respondents were asked to complete the anonymous questionnaire within 2 weeks. We assessed objective understanding and perceived subjective understanding of IC using a Japanese version of the Quality of Informed Consent scale (QuIC). Then we analyzed the characteristics of women whose understanding was poor, and clarified the association between providing information and their understanding of the study protocol.

**Results:**

The average QuIC scores were 78.2 and 82.2 (out of 100 each) for objective and subjective understanding, respectively. These are generally acceptable scores for participants’ understanding of an RCT. However, there were four domains with low scores, indicating poor understanding: (1) experimental nature of the study, (2) potential risks or discomfort, (3) benefit to self, and (4) compensation.

**Conclusions:**

Healthy volunteers generally well understood the J-START. Nevertheless, there were some domains in need of improvement. In order to facilitate participants’ understanding, it is necessary to provide training to reduce differences in information-providing procedures between medical centres and to endeavour to provide consistent information and conditions.

**Trial registration:**

The J-START was registered with the University Hospital Medical Information Network Clinical Trial Registration (UMIN-CTR), Japan (registration number: UMIN000000757), on July 1, 2007.

## Background

It has been suggested that the complex design of a clinical trial, such as a randomized controlled trial (RCT), is difficult to understand for participants. Although participants are enrolled in a trial only after undergoing the informed consent (IC) process, their understanding of the trial in which they are participating is often insufficient [1–9]. The following two factors might account for this insufficient understanding. First, participants cannot distinguish already established, evidenced examination and treatment procedures from study protocols necessitated by treatment that has not yet been established [1–5]. Second, it is difficult to understand the concepts that are characteristic of RCTs, such as randomization and placebos [5–9].

To our knowledge, no large-scale RCT with more than 70,000 healthy volunteers from the general population had been conducted in Japan before the Japan STrategic Anti-cancer Randomized controlled Trial (J-START) was initiated [10]. The J-START is an RCT on the effectiveness of ultrasonography in breast cancer screening that was developed to assess breast cancer screening in Japan in 2007.

According to a survey conducted in Japan on the attitudes of the healthy general population toward clinical trials [11], participation in them is widely recognized as a volunteer activity that provides an opportunity to contribute to society. However, it has been reported that participants do not sufficiently understand the definitions and contents of clinical trials [11]. There are no reports on the understanding of an RCT targeting a general population of healthy women in Japan. Moreover, there are guidelines but no laws about clinical trials except for pharmaceutical products in Japan. Therefore, as a matter of ethics, clinical trials conducted in Japan should make an effort to provide understandable ICs for eligible participants. However, there is no validity of the ICs provided in Japan with respect to ethics compared with trials in other countries. Thus, we surveyed the degrees of objective and subjective understanding of the IC process among J-START participants. Additionally, factors associated with degrees of understanding were investigated. This is a survey study of the quality of IC given to participants of an RCT to clarify what would be helpful for future trials.

## Methods

### Overview of J-START

J-START is a multi-centre RCT with 42 participating medical centres in 23 prefectures across Japan. It is supported by science research funds from the Japan Ministry of Health Labour, and Welfare. The aim of the J-START is to assess the effectiveness of screenings for breast cancer among women in their 40s. As of 31 March 2011, 76,196 healthy female volunteers had enrolled [12]. The primary endpoints were sensitivity, specificity, and advanced cancer ratio in both screening groups: the intervention group (women who received ultrasonography in addition to mammography) and the control group (women who received mammography only). The research coordinators verbally explained the purpose and method of the RCT to all eligible participants while collecting ICs.

J-START participants were recruited by trained research coordinators. Potential participants were given a supplementary leaflet by mail before visiting the medical centres. At the medical centres, an educational video recording of the study was presented and a verbal description and a booklet were provided for IC. The total time of the educational video was almost 7 minutes, and it played continuously in the visitors’ waiting rooms of the medical centres.

### Participants and study methods

Of the 42 medical centres participating in J-START, five medical centres in Iwate, Yamagata, Tokyo, Okayama, and Okinawa participated in our study. From 10 November 2010 to 31 January 2011, we distributed anonymous questionnaires to newly enrolled participants regarding their understanding of the RCT at the five medical centres in different Japanese prefectures. At the time we received approval for this survey in September 2010, only the above-cited medical centres were recruiting new participants; furthermore, only those centres had the proper personnel for conducting the accompanying research.

We distributed the anonymous questionnaires regarding participants’ understanding of the RCT at the medical centres. Informed consent for participating in our investigation of participants’ understanding of the RCT was assumed by response to the questionnaire. The set of documents distributed to each participant included a letter describing the purpose and method of the study, the questionnaire, and a return envelope. Ethical approval was obtained from the Institutional Review Board of Tohoku University Graduate School of Medicine on 27 September 2010 (No. 2010–279).

### Questionnaire

Objective understanding (actual knowledge) and subjective understanding (self-assessed understanding) were measured using a Japanese version of the Quality of Informed Consent (QuIC), which is a scale established in the United States to assess participants’ knowledge and self-assessment of clinical trials [6]. The items cover the basic elements regarding the protection of human participants as stated in US federal regulations (45CFR46) [13]. In addition to English [6, 14–16], the QuIC has been translated into French [17] and Swedish [18]. It has been used not only in clinical trials for developing anticancer agents but also in studies on social support [19] and in a genome cohort study [14].

Part A of the Japanese version of the QuIC contains 16 items measuring actual knowledge of the basic elements of IC. The total QuIC Part A score represents an average of the 16 items on objective understanding, ranging from 0 (lowest) to 100 (highest) [6]. Each item is measured with a triple-bounded binary-choice format: “Totally agree”, “Unsure”, and “Strongly disagree”. The points were given as follows: 100 points for the correct answer, 50 points for “Unsure”, which is neither correct nor incorrect, and 0 points for a wrong answer. Answers without a response were marked “No reply”. There were four negatively-phrased items, for which the correct answer was “Disagree”, so as to avoid agreement bias that might result from all correct answers being “Agree” [6].

Part B of the Japanese version of the QuIC consists of 14 items assessing subjective understanding of important elements of the trial. The total score of the QuIC Part B is an average of the scores for the 14 items [6]. Each item was measured with a quintuple-bounded binary-choice format: 100 points for “Understood very well”, 75 points for “Generally understood”, 50 points for “Neither understood nor did not understand”, 25 points for “Almost did not understand”, and 0 points for “Did not understand”.

The J-START is an RCT that enrolled healthy volunteers as the participants for breast cancer screening. For that reason, the QuIC questions that pertained only to Phase I clinical studies were deleted (in the original QuIC full version: questions A6, A7, A8, and A10). We then had one Japanese and one Chinese healthcare worker who use English and Japanese on a daily basis translate the remaining questions into Japanese sentences that would be readily understandable in the context of current Japanese culture. Next, we had back-translated that Japanese into English by a different person who used English and Japanese on a daily basis. Finally, that back-translated version was checked by a person whose native language was English. The comprehensibility of the technical terms and expressions in not only the Japanese QuIC, but also the full questionnaire including original items, was checked by having a medical ethics specialist and medical staffers complete the entire questionnaire. We then made revisions to the materials based on their replies. To ensure the validity of the full questionnaire, we evaluated the materials’ reliability by test-retest as following. We asked the new participants in J-START, which has the same conditions as the present study, to answer the full questionnaire, including the Japanese version of QuIC and other items, twice, at a 2-week interval. Twenty-one participants answered all the questions in the questionnaire twice. Weighted kappa statistics for QuIC ranged from 0.32 (objective risks) to 0.67 (subjective experimental nature of study), respectively. Cronbach’s alpha values for QuIC for internal consistency were 0.57 and 0.89 for Parts A and B, respectively.

### Other questionnaires

We administered additional questionnaires to measure age, educational level, marital status, work status, and study allocation of the RCT. These also assessed impressions of materials provided and prior knowledge about J-START and RCTs. These questionnaires and the Japanese QuIC were validated by the pilot study at the same time.

Multimedia information materials had been prepared for J-START (for example, leaflets, educational DVDs). Their effectiveness was assessed with quintuple-bounded binary-choice items (“Very helpful”, “Almost helpful”, “Neither helpful nor unhelpful”, “Almost unhelpful”, and “Not at all helpful”). In addition, alternative responses were prepared as follows: “Have not read”, and “Have not watched”.

The effectiveness of the verbal description during IC was assessed with the following five questions: (1) “The research coordinator facilitated your understanding”. (2) “You had an opportunity to ask questions”. (3) “You had sufficient time to understand the process”. (4) “It was easy to say ‘no’, if you did not want to continue your participation”. (5) “You sought further explanation of the study”. The answer to these five questions was coded as follows: 1 for “Yes” and 0 for “No” or “Unsure”.

### Statistical analysis

Continuous variables are reported with means and standard deviations, whereas categorical variables are reported as percentages. Bivariate correlations were conducted between the QuIC scores and each independent variable. Correlations were assessed using chi-squared tests. All analyses were conducted using SAS statistical software (version 9.3; SAS Institute Inc., Cary, NC, USA).

## Results

### Participant characteristics

Participant characteristics are shown in Table 1. Of the 745 questionnaires distributed, 376 (50.4%) were returned. Among 376 survey respondents, almost 70% graduated from vocational school, junior college, or above. There were 280 married participants (74.5%), and 308 participants (81.9%) were employed. In relation to group allocation of the RCT, 198 participants (52.7%) were assigned to the intervention group, and 162 participants (43.1%) were assigned to the control group.

**Table 1:** **Characteristics of the study participants**

		N	%
Education level	Junior high school	3	0.8
	High school	96	25.5
	Junior college*	154	41.0
	University, graduate school	105	27.9
	No reply	18	4.8
Marital status	Married, living with a partner	280	74.5
	Unmarried, widow, divorced	78	20.7
	No reply	18	4.8
Work status	Employed	308	81.9
	Unemployed	49	13.0
	No reply	19	5.1
Study allocation**	Intervention group	198	52.7
	Control group	162	43.1
	No reply	16	4.3

The mean age of the participants was 43.8 years (SD = 3.1). *Junior college includes vocational school and technical college. **Participants were randomly assigned to the intervention group (screening by mammography and ultrasound) or control group (screening only by mammography) in the Japan STrategic Anti-cancer Randomized controlled Trial (J-START).

### Objective understanding

The results of the objective understanding (QuIC Part A) are shown in Table 2. The average QuIC Part A score was 78.2 (95% CI, 68.6 to 87.8). Participants generally understood most question items well. However, the accuracy rates for some items were low. Specifically, there were accuracy rates of 14.6% for Question A4 on the “Experimental nature of study”, 14.1% for Question A8 on “Potential risks or discomforts”, 34.6% for Question A9 on “Benefit to self”, and 33.0% for Question A13 on “Compensation”.

**Table 2:** **Percentage of participants by QuIC Part A score: an objective understanding of J-START**

		QuIC items	QuIC scores			No
			1	2	3	reply
A1.	Nature of research	When I signed the consent form for J-START, I knew that I was agreeing to participate in a clinical trial	0.3	5.6	92.6*	1.6
A2.	Purpose of research	The main goal of J-START is improving breast cancer screening for future generations	0.0	2.7	95.7*	1.6
A3.	Duration of procedures	I have been informed about the duration of J-START	4.8	30.3	63.3*	1.6
A4.	Experimental nature of study	All tests in J-START are standardized	14.6*	26.1	57.5	1.9
A5.	Purpose of research	The major purpose of J-START is to assess the effectiveness of ultrasound screening for breast cancer among Japanese women aged 40–49 years	1.1	10.6	86.7*	1.6
A6.	Purpose of research	Neither the mammography screening nor the combined use of mammography and ultrasound screening have been proven as the best screening method for Japanese women aged 40–49 years	0.8	25.3	72.3*	1.6
A7.	Procedures to be followed	After I agreed to participate in J-START, my examination was chosen randomly between mammography screening or combined use of mammography and ultrasound screening	1.9	11.4	85.1*	1.6
A8.	Potential risks or discomforts	Compared with standard breast cancer screening, J-START does not carry any additional risks or discomforts	14.1*	35.6	48.7	1.6
A9.	Benefits to self	I might not receive any direct medical benefits from my participation in J-START	25.5	38.3	34.6*	1.6
A10.	Benefits to others	By participating in J-START, I am helping the researchers gather information that might benefit future breast cancer screening procedures	0.3	10.4	87.8*	1.6
A11.	Confidentiality	Because I am participating in a clinical trial, it is possible that the study sponsor, various government agencies, or others who are not directly involved in my care will review my medical records	3.7	33.2	61.4*	1.6
A12.	Alternatives to participation	My doctors did not offer me any alternative breast cancer screening procedures beyond J-START.	87.8*	9.3	1.3	1.6
A13.	Compensation	The consent form I signed indicates who will pay for treatment if I am injured or become ill as a result of participation in this clinical trial	14.9	50.3	33.0*	1.9
A14.	Study contacts	The informed consent form listed study contact persons	4.8	19.7	73.7*	1.9
A15.	Voluntary nature of participation	If I had not wanted to participate in this clinical trial, I could have declined to sign the consent form	1.1	11.4	85.6*	1.9
A16.	Voluntary nature of participation	I must remain in the clinical trial, even if I decide that I would like to withdraw someday	60.1*	30.1	8.2	1.6

Quality of Informed Consent (QuIC) is a scale for assessing participants’ understanding of clinical trials. Part A assesses objective understanding and Part B assesses subjective understanding. QuIC Part A possible responses are 1 (quite disagree), 2 (unsure) and 3 (totally agree) [6]. *Correct answer. J-START, Japan STrategic Anti-cancer Randomized controlled Trial.

### Subjective understanding

The results of the subjective understanding (QuIC Part B) are shown in Table 3. The average QuIC Part B score was 82.2 (95% CI, 69.3 to 95.1). However, for Question B11 on “Compensation”, “Who will pay for treatment if you are injured or become ill because of participation in J-START?”, the combined percentage of participants who indicated that “I understood very well” and “I generally understood” was only 44.7%. For Question B6 on “Potential risks or discomforts”, the total percentage of participants who indicated that “I understood very well” and “I generally understood” was 78.2%. This question item corresponded to Question A8 on “Potential risks or discomforts”. It is worth noting that, although objective understanding of this item was low, subjective understanding was high.

**Table 3:** **Percentage of participants by QuIC Part B score: a subjective understanding of J-START**

		QuIC questions	QuIC scores					No
			1	2	3	4	5	reply
B1.	Nature of research	This breast cancer screening involves a research component	1.1	2.1	3.7	39.6	51.6	1.9
B2.	Purpose of research	What the researchers are trying to understand in J-START	0.3	1.1	1.6	36.7	59.0	1.3
B3.	Duration of procedures	How long you will be in J-START	1.9	7.2	9.0	33.8	46.8	1.3
B4.	Procedures to be followed	The tests and procedures you will undergo	1.1	0.8	2.7	30.6	62.2	2.7
B5.	Experimental nature of study	Which of these tests and procedures are experimental	1.1	4.3	7.5	38.6	39.4	9.3
B6.	Potential risks or discomforts	The potential risks or discomforts associated with participating in J-START	1.9	4.5	13.8	34.8	43.4	1.6
B7.	Benefits to self	The potential benefit to you for participating in J-START	2.1	1.9	14.4	42.0	37.8	1.9
B8.	Benefits to others	How your participation in this clinical trial might benefit future patients	0.3	0.3	2.4	31.4	64.1	1.6
B9.	Alternatives to participation	The alternative to participation in the clinical trial	0.8	1.3	6.4	28.5	61.4	1.6
B10.	Confidentiality	The effect of clinical trial participation on the confidentiality of your medical records	0.5	0.0	3.2	30.1	64.9	1.3
B11.	Compensation	Who will pay for treatment if you are injured or become ill due to participation in J-START	14.9	19.4	18.4	21.8	22.9	2.7
B12.	Study contacts	Whom you should contact if you have questions or concerns regarding J-START	4.5	6.4	11.7	31.9	43.9	1.6
B13.	Voluntary nature of participation	The fact that participation in J-START is voluntary	0.5	0.3	0.3	17.8	79.8	1.3
B14.	Overall	Overall, how well did you understand your specified clinical trial when you signed the consent form?	0.8	2.4	4.5	65.7	25.3	1.3

Quality of Informed Consent (QuIC) is a scale for assessing participants’ understanding of clinical trial procedures. Part A assesses objective understanding and Part B assesses subjective understanding [6]. QuIC Part B responses were as follows: 1 for “did not understand”, 2 for “almost did not understand”, 3 for “neither understood nor didn’t understand”, 4 for “generally understood”, and 5 for “understood very well”. J-START, Japan STrategic Anti-cancer Randomized controlled Trial.

### Assessment of explanatory materials and information-providing procedures

In order to examine how various information-providing procedures used during the IC process related to participants’ understanding, we asked participants about their prior knowledge and asked them to give their impression of the materials used for explanation and verbal description, and to evaluate each procedure (Table 4). Although informational leaflets explaining the trial had been mailed to the participants in advance, 279 (74.2%) indicated that they did not have any prior knowledge of J-START. The participants highly valued the informational leaflets and the educational videos explaining the trial. These were regarded as “Helpful for understanding” by 266 (70.7%) and 277 participants (73.7%), respectively. Three hundred and forty-six participants (92.0%) reported that their understanding had been confirmed by the research coordinator at the end of the oral description during the IC process. It would be expected that, at the time of that interaction, the research coordinator would become cognizant of the participant’s questions and points of confusion, but we do not know what sort of communication took place later in this study. Only 10 participants (2.7%) indicated that they sought further explanation of the trial at that time.

**Table 4:** **Questionnaire on participants’ impression of the informed consent process**

Item	Categories	N	%
**Prior knowledge**			
Did you know about “RCTs” before participating in J-START?	Yes	108	28.7
	No	268	71.3
Did you know about J-START before the informed consent procedure?	Yes	97	25.8
	No	279	74.2
**Helpfulness of media**			
Did the information leaflet help you understand J-START?	Helpful	266	70.7
	Unsure	62	16.5
	No	48	12.8
Did the educational video help you understand J-START?	Helpful	277	73.7
	Unsure	25	6.7
	No	74	19.7
**Evaluations of the verbal delivery of the information during the informed consent procedure**			
Did the research staff confirm your understanding?	Yes	346	92.0
	Unsure	17	4.5
	No	9	2.4
	Missing	4	1.1
Did you need further information?	Yes	10	2.7
	Unsure	58	15.4
	No	299	79.5
	Missing	9	2.4
I had sufficient opportunity to ask questions	Yes	338	89.9
	Unsure	14	3.7
	No	20	5.3
	Missing	4	1.1
I had enough time to understand information	Yes	259	68.9
	Unsure	102	27.1
	No	12	3.2
	Missing	3	0.8
Did you find it easy to refuse participation?	Yes	285	75.8
	Unsure	60	16.0
	No	23	6.1
	Missing	8	2.1

J-START, Japan STrategic Anti-cancer Randomized controlled Trial; RCT, randomized controlled trial.

### Factors associated with low objective understanding

Table 5 shows the association between factors at the time of the IC process and the four objective understanding items with low accuracy rates; that is, A4, A8, A9, and A13 of QuIC Part A. The medical centre showed a correlation with two questions: Question A8 on “Potential risks or discomforts” (*P* = 0.009) and Question A13 on “Compensation” (*P* < 0.0001). The existence of prior knowledge of the RCT or J-START itself was statistically significant only for Question A4 on “Experimental nature of study”, (RCT, *P* = 0.002; J-START, *P* = 0.003). The educational video showed a correlation with the two questions A8 on “Potential risks or discomforts” (*P* = 0.003) and A13 on “Compensation” (*P* = 0.001). Sufficient opportunity to ask a question showed correlations with Question A8 on “Potential risks or discomforts” (*P* = 0.025), Question A9 on “Benefit to self” (*P* = 0.023), and Question A13 on “Compensation” (*P* = 0.049), while enough time to achieve understanding showed correlations with Question A8 on “Potential risks or discomforts” (*P* = 0.007) and Question A13 on “Compensation” (*P* = 0.013). The atmosphere at the venue when the decision to participate was made showed correlations with Question A9 on “Benefit to self” (*P* = 0.001) and Question A13 on “Compensation” (*P* = 0.043), but for each case there was a low percentage of participants who felt the atmosphere made it hard to refuse to participate. No statistically significant correlation was found between the degree of understanding and the following factors: education level, marital status, work status, informational leaflet, whether the research staff confirmed whether the participant understood, or whether the participant wanted to ask further questions at the time of IC.

**Table 5:** **Factors associated with low comprehension scores of QuIC Part A items**

	A4. Experimental nature of study				A8.Potential risks or discomforts				A9. Benefit to self				A13. Compensation			
	Quite disagree*	Unsure	Totally agree	***P***	Quite disagree*	Unsure	Totally agree	***P***	Quite disagree	Unsure	Totally agree*	***P***	Quite disagree	Unsure	Totally agree*	***P***
**Medical centres**																
Site A	3.5%	7.1%	12.7%	0.54	3.0%	6.5%	13.8%	0.009	5.7%	8.7%	8.9%	0.927	0.8%	6.5%	16.0%	<0.0001
Site B	1.1%	3.3%	5.4%		2.7%	4.1%	3.0%		3.2%	3.5%	3.0%		2.2%	6.0%	1.6%	
Site C	6.5%	9.2%	22.2%		4.3%	14.3%	19.2%		9.2%	14.6%	14.1%		7.6%	21.7%	8.7%	
Site D	3.0%	4.1%	8.9%		3.5%	6.8%	5.7%		3.8%	7.0%	5.1%		1.6%	9.8%	4.6%	
Site E	0.8%	3.0%	9.2%		0.8%	4.6%	7.8%		4.1%	5.1%	4.1%		3.0%	7.3%	2.7%	
**Prior knowledge: had you known about “RCTs” before you took part in J-START?**																
Yes	6.2%	9. 8%	13.0%	0.002	5.4%	10.5%	13.0%	0.266	6.5%	11.9%	10.5%	0.613	4.1%	16.0%	8.9%	0.629
No	8.7%	16.8%	45.5%		8.9%	25.7%	36.5%		19.5%	27.0%	24.6%		11.1%	35.2%	24.7%	
**Prior knowledge: had you known about J-START before the informed consent?**																
Yes	6.5%	7.1%	12.5%	0.003	3.5%	8.7%	13.8%	0.703	6.2%	11.4%	8.4%	0.529	4.1%	13.8%	8.1%	0.851
No	8.4%	19.5%	46.1%		10.8%	27.6%	35.7%		19.7%	27.6%	26.8%		11.1%	37.4%	25.5%	
**Helpfulness of media: Educational video was an aid to understanding J-START**																
Yes	9.8%	19.8%	44.2%	0.538	9.5%	24.6%	39.5%	0.003	19.2%	27.0%	27.3%	0.517	9.2%	35.5%	29.0%	0.001
No	1.1%	1.4%	4.1%		0.3%	4.3%	2.2%		1.4%	3.5%	1.9%		1.1%	4.3%	1.1%	
Unsure	4.1%	5.4%	10.3%		4.6%	7.3%	7.8%		5.4%	8.4%	6.0%		4.9%	11.4%	3.5%	
**Evaluations of the verbal delivery of the information: I had sufficient opportunity to ask questions**																
Yes	14.1%	23.9%	51.8%	0.411	14.1%	30.8%	44.9%	0.025	25.1%	33.5%	31.1%	0.023	13.3%	44.4%	32.0%	0.049
No	0.8%	2.7%	6.8%		0.3%	5.4%	4.6%		0.8%	5.4%	4.1%		1.9%	6.8%	1.6%	
**Evaluations of the verbal delivery of the information: I had enough time to understand information**																
Yes	9.8%	17.9%	40.7%	0.828	9.2%	21.6%	37.6%	0.007	18.1%	25.7%	24.6%	0.729	9.2%	32.8%	26.3%	0.013
No	5.2%	8.7%	17.9%		5.1%	14.6%	11.9%		7.8%	13.2%	10.5%		6.0%	18.4%	7.3%	
**Evaluations of the verbal delivery of the information: Did you find it easy to say no?**																
Yes	11.7%	18.7%	45.0%	0.409	10.5%	25.4%	39.5%	0.137	22.4%	25.7%	27.3%	0.001	13.0%	36.0%	26.3%	0.043
No	3.3%	7.9%	13.6%		3.8%	10.8%	10.0%		3.5%	13.2%	7.8%		2.2%	15.2%	7.3%	

*Correct answer. J-START, Japan STrategic Anti-cancer Randomized controlled Trial; RCT, randomized controlled trial.

## Discussion

The present study aimed to evaluate the degree of participants’ understanding of an RCT and investigate the associated factors. We administered questionnaires to 376 healthy Japanese women on the day of enrolment at five study sites using a Japanese version of the QuIC. Although healthy volunteers generally well understood J-START, there were some domains in need of improvement. Until the present study, there have been no reports on either the understanding of an RCT targeting the general population of healthy women or reports concerning evaluations using international and validated scales in Japan. The present study has demonstrated hints of improvement in the IC process in an RCT that targeted the general population of healthy people. The participants generally provided correct responses to the majority of the questions. Furthermore, the QuIC scores in the present survey were comparable to the scores in the preceding studies [14–16, 18]. The results of the present study revealed a tendency for the degree of subjective understanding to be higher than the degree of objective understanding, which is similar to the findings of earlier studies. The higher degree of subjective understanding can be thought to be related to the ease of understanding the IC and/or a feeling of satisfaction with the amount of information, but the reason for discrepancy with the degree of objective understanding warrants further study. Thus, the purpose of IC seems to have largely been achieved. However, among the items specified by the US Federal Regulation (Chapter 45, Part 46) that should be explained to individuals eligible for participation in a clinical trial, “Experimental nature of study”, “Potential risks or discomforts”, “Benefit to self”, and “Compensation” were not correctly understood by some participants. In previous studies, “Experimental nature of study” [1, 8, 14, 18], “Potential risks or discomfort” [8, 14, 18, 20],“Benefit to self” [5], and “Compensation” [14, 18] were reported to be difficult for participants to understand. Therefore, strategies need to be developed to facilitate better understanding of these items among participants.

It is necessary to consider the current status of breast cancer screening in Japan, giving consideration to these three factors: “Experimental nature of the study”, “Potential risks or discomforts”, and “Compensation”. Regarding “Experimental nature of the study” and “Potential risks or discomforts”, because of the comfort level and familiarity derived from the fact that ultrasonography is a well-known method, participants are unlikely to understand that a trial is conducted due to the lack of any established intervention, and that there are potential risks or discomforts involved. Moreover, regarding “Compensation”, participants might not have paid attention to the explanation of compensation for any problems about intervention. Mammography, which is currently used for breast cancer screening, is known to sometimes cause pain from the compression of a breast, as well as minor exposure to x-rays during imaging. Ultrasonography is generally perceived to be more comfortable than mammography. Therefore, it might be difficult to imagine that health problems could be caused by ultrasonography. Moreover, because breast ultrasonography has already been employed as a breast cancer screening method for opportunistic screening (for example, complete physical examination), a perception of this procedure as a well-established screening method might not reflect the true situation. In fact, scientific evidence for breast ultrasonography as a method for mass screening has remained under investigation in J-START since 2007. Because the procedure has been employed as an effective screening method in routine practice in breast surgery departments, the present survey might have included participants who had previously undergone ultrasonography as a component of their medical care.

There were three possible reasons for participants’ misunderstanding of IC, which have been referenced in previous studies. First, the explanatory materials might have been difficult to understand [21, 22]. Second, the comprehension ability of participants might have been insufficient [4, 23]. Third, the information-providing procedures used during the IC process might have varied between medical centres [9].

First, in relation to difficult explanatory materials, well-considered wording [22] and explanations using written, paper-based information (for example, leaflets and booklets) appear to improve understanding of RCTs [21]. Further, it is preferable to provide information with a combination of several materials [24]. When we prepared the informational leaflets and the educational video, we sought to use simple words and considered a combination of materials. The resultant material was also approved by the institutional ethical review board. As shown in Table 4, the majority of the participants indicated that the explanatory materials were “Helpful for understanding”. This demonstrates that the explanatory materials were perceived to be sufficiently easy to understand. Table 5 indicates that prior knowledge influenced misunderstanding of “Experimental nature of the study”, but prior knowledge had not always been given by our leaflet, and the educational video did not help correct misconceptions. In a study simulating a situation in which children and their parents undergo the IC process, it was reported that multimedia materials with visual and auditory information are preferable to paper-based information and could be expected to improve understanding, particularly among parents [25]. Other studies have indicated that a simplified IC form with emphasis added by verbal description [26], and user testing, which examines not only the wording of leaflets, but also the layout and paper thickness, in order to design interesting materials for participants to read, leads to improved explanatory materials and better understanding [27]. However, in systematic reviews of understanding of RCTs [28] and of trials and ICs [29], it was reported that multimedia informational materials were not as effective for improving participants’ understanding as test-feedback quizzes or discussion of the IC process [28, 29]. The result of our survey corroborated the systematic reviews. Improving face-to-face communication would foster better understanding than improving written materials.

Second, regarding the comprehension ability of participants, approximately 70% of the participants in the present study graduated from vocational school, junior college, or above (Table 1). Because the illiteracy rate in Japan is low due to the 9 years of mandatory education, insufficient comprehension ability is an unlikely explanation.

Regarding the third possible reason for participants’ misunderstanding of IC, there were, unfortunately, significant gaps or inconsistencies between the IC information-providing procedures of the different medical centres (Table 5). Regrettably, we did not have the data on how IC was obtained from each of the participants. For the standardized information dissemination process in J-START, the manual included specific procedures concerning IC and the use of materials, and held several training sessions for the research coordinators, who had previous experience working at a medical centre (for example, nurses and public health nurses). To avoid misconceptions arising from verbal communication, explanatory materials were also integrated into the IC process. Despite this, a certain number of participants reported as being unsure about these materials. However, we did not have the IC process data, and it was not clear that the process was lost or the participants did not read or watch. Given that explanatory materials are reported to improve participants’ understanding [8], the differences in understanding observed in the present study might be attributable to insufficient use of the materials. A previous systematic review reported that having a long discussion time with a team member and a neutral educator was effective for participants’ understanding, and it worked even over the telephone [29]. In J-START, this type of discussion was not programmed officially. If we could have had the opportunity to include such a discussion, IC and accompanying materials might have been delivered more effectively. In order to more effectively perform the IC process, we must create opportunities for the team members to have frequent discussions with the educator, with the topic of discussion to include better information-providing procedures.

A limitation of the present study was that not all of the J-START study sites were included. However, the validity of the QuIC was evaluated and the reliability of the questionnaire was tested. Significantly more participants in the intervention group returned the questionnaires than in the control group. Therefore, the present findings were not derived from random sampling with respect to which participants received an explanation of J-START. Unfortunately, we had to use the most convenient sampling method, and could not collect data on non-responders without authorization.

## Conclusions

Healthy volunteers generally well understood the RCT in which they participated. The results of the present study suggest that when an RCT on a minimally invasive intervention is conducted, both the researchers who provide explanations and the participants might neglect the following four items: the fact that interventions under investigation have not been standardized, the possible benefits and disadvantages of participating in the trial, and compensation in case of injury resulting from participation. In order to facilitate participants’ understanding, it is necessary to provide training for the research team members to reduce differences in information-providing procedures between medical centres and to endeavour to provide consistent information and conditions.

## Abbreviations

IC: informed consent
J-START: Japan STrategic Anti-cancer Randomized controlled Trial
QuIC: Quality of Informed Consent
RCT: randomized controlled trial.
